# The correlation between Jun N-terminal kinase pathway-associated phosphatase and Th1 cell or Th17 cell in sepsis and their potential roles in clinical sepsis management

**DOI:** 10.1007/s11845-020-02382-5

**Published:** 2020-10-20

**Authors:** Dan Yu, Xiaohong Peng, Peng Li

**Affiliations:** Department of Anesthesiology, Wuhan Fourth Hospital, Puai Hospital, Tongji Medical College, Huazhong University of Science and Technology, Jiefang Avenue, Gutiansan Road, Wuhan, 430000 People’s Republic of China

**Keywords:** Inflammatory cytokines, Jun N-terminal kinase (JNK) pathway-associated phosphatase, Mortality, Sepsis, T helper type 1, T helper type 17

## Abstract

**Background:**

We aimed to investigate the association between Jun N-terminal kinase (JNK) pathway-associated phosphatase (JKAP) and T helper type 1 (Th1) cell or Th17 cell, and their clinical values in sepsis patients.

**Methods:**

Totally 125 sepsis patients and 100 healthy subjects as controls were included. Peripheral blood was extracted from each sepsis patient and each control, then serum and peripheral blood mononuclear cell (PBMC) were separated. JKAP and inflammatory cytokines were detected in serum by ELISA; Th1 cell or Th17 cell proportion was detected in PBMC using flow cytometry.

**Results:**

JKAP level was downregulated while Th1 and Th17 cell proportions were upregulated in sepsis patients compared with controls. JKAP level negatively correlated with Th1 cell proportion in sepsis patients and controls, while was only negatively associated with Th17 cell proportion in sepsis patients but not in controls. In sepsis patients, JKAP level negatively associated with TNF-α, IL-1β, and IL-17 expressions. Meanwhile, JKAP level negatively but Th17 cell proportion positively correlated with acute physiology and chronic health evaluation II (APACHE II) and sequential organ failure assessment (SOFA) scores; however, Th1 cell proportion only positively associated with APACHE II score but not SOFA score. Additionally, JKAP level was reduced, while Th1 and Th17 cell proportions were increased in septic deaths compared with survivors. Multivariate logistic regression model disclosed that JKAP level and Th17 cell proportion independently predicted 28-day mortality.

**Conclusion:**

Blood JKAP correlates with decreased Th1 and Th17 cells, also associates with reduced inflammatory cytokines, disease severity, and favorable outcome in sepsis patients.

## Introduction

Sepsis, an ancient disease lasting for thousands of years, is described as a lethal systemic disease resulting from the aberrant host response to infection of various origins, which could finally lead to a multiple organ dysfunction syndrome (MODS) [1]. Every year, there are millions of people attacked by sepsis and dead because of it, which has made sepsis a predominant cause of worldwide mortality [2]. However, for those who survive from sepsis, they have to confront different outcomes post hospitalization, such as a permanent health damage (e.g., cognitive injury), aggravation of health status, and even death caused by an exacerbation of clinical sequelae [3]. Sepsis management, based on the goal of preventing death in short-term, mainly composes of anti-infection, resuscitation, and vasopressor for patients with hypotension or abnormal lactate level [4]. What is more important, development in sepsis management is rather unfavorable despite recent progress in modern medicine, for instance, an effective drug used for advanced stage sepsis, Drotrecogin alfa (activated), is only applied in the market for several years before its abandonment in 2011 [5, 6]. Hence, there is a desperate need in finding more effective methods to improve the management of sepsis.

Jun N-terminal kinase (JNK) pathway-associated phosphatase (JKAP) is a tyrosine phosphatase that specifically acts on JNK kinase; it not only dephosphorylates the JNK kinase but also regulates many other pathways [7, 8]. Previously, a research reveals a potential role of JKAP in serving as a biomarker that is related to disease severity, inflammation, and patients’ survival in patients with sepsis [9]. Th1 cell and Th17 cell, differentiated from CD4^+^ T cells activated by different categories of cytokines, are increasingly indicated to participate in the regulation of infection and inflammation-related diseases, including sepsis [10–13]. It has been reported that JKAP regulates the functions of T cells, for instance, JKAP can inhibit the CD4^+^ T cell activation and differentiation to Th1 and Th17 cells in peripheral blood mononuclear cell (PBMC) collected from patients with active inflammatory bowel disease (IBD) [14]. Another study reveals that JKAP level in T cells is correlated with disease severity and renal outcome in patients with systemic lupus erythematosus (SLE) [15]. Thus given the regulatory roles of JKAP, Th1 cell and Th17 cell in sepsis, along with the modulating role of JKAP in CD4^+^ T cell activation and differentiation to Th1 or Th17 cell, we presumed that JKAP may be correlated with the Th1 cell or Th17 cell proportion in sepsis patients, and the three of them possibly have potential assisting in the management of sepsis. However, their intercorrelations and values regarding disease management remain elusive in sepsis patients.

Therefore, the aim of the present study was to investigate the association between JKAP and Th1 cell or Th17 cell, and their correlations with inflammatory cytokines, disease severity as well as 28-day mortality in sepsis patients.

## Methods

### Study subjects

Between November 2017 and September 2019, 125 sepsis patients admitted to our hospital were consecutively enrolled. The diagnosis of sepsis referred to the Third International Consensus Definitions for Sepsis and Septic Shock [16]. All sepsis patients recruited in this study were above 18 years old and admitted to our department within 12 h after sepsis onset. The sepsis patients who were complicated by other fatal diseases (e.g., hematologic malignancies, solid tumors, acquired immune deficiency syndrome) or received immunosuppressive therapy before admission were excluded. In addition, 100 healthy subjects who underwent healthy examination in the hospital between October 2019 and December 2019 were enrolled as controls. The controls had age and gender matched with the sepsis patients, no obvious abnormalities in biochemical indexes, and no history of hematological malignancies, solid tumors, sepsis, or other severe infections. This study was approved by the Institutional Review Board of our hospital. Written informed consents were provided by all subjects or their family members. In addition, the treatment of patients was not delayed by any process of this study.

### Sample collection

A total of 3 mL peripheral blood was extracted from each sepsis patient at admission, and 3 mL peripheral blood was collected from each control subject on enrollment. After collection, 1.5 mL peripheral blood was immediately processed with gradient density centrifugation to isolate PBMC, and another part of peripheral blood was centrifuged at 3000*g* for 15 min to separate serum samples. Then, the PBMC was stored at 4 °C and the serum was stored at − 80 °C until further detection.

### Flow cytometric analysis of Th1 and Th17

For sepsis patients and controls, the profiling of Th1 and Th17 in PBMC samples was performed using Human Th1 or Th17 Phenotyping Kit (BD Pharmingen™, Franklin Lake, USA), and the procedure was carried out within 24 h after PBMC isolating based on the guidance of instruction. Then, Th1 cell proportion and Th17 cell proportion were counted by FACSARIA II flow cytometer (BD, Franklin Lake, USA).

### JKAP and inflammatory cytokines detection

For sepsis patients and controls, the levels of JKAP in serum samples were detected by enzyme-linked immunosorbent assay (ELISA). For sepsis patients, the levels of inflammatory cytokine (tumor necrosis factor-α (TNF-α), interleukin-1β (IL-1β), and interleukin-17 (IL-17)) in serum samples were detected by ELISA as well. The ELISA kits used in this assay were as follows: human JKAP ELISA Kit (Shanghai Enzyme-linked Biotechnology Co., Ltd, Shanghai, China), human TNF-α ELISA Kit (Thermo Fisher Scientific, Waltham, USA), human IL-1β ELISA Kit (Thermo Fisher Scientific, Waltham, USA), human IL-17 ELISA Kit (Thermo Fisher Scientific, Waltham, USA). All the procedures were carried out according to the instructions. In addition, the process of ELISA assay was as follows. In brief, firstly, samples and standard solutions were added to the coated 96-well plate. Then, the plate was covered and incubated for 2 h at room temperature on a plate shaker at 400 rpm. After washing 4 times with wash buffer, a prepared biotin conjugate was added to the wells, and the plate was incubated for 1 h at room temperature on a plate shaker at 400 rpm. Subsequently, the streptavidin-HRP solution was added to the wells after they were washed 4 times with wash buffer. Then, the plate was incubated for 45 min at room temperature on a plate shaker at 400 rpm. Afterward, tetramethylbenzidine substrate was added to the wells and incubated for 10 min in the dark on a plate shaker at 400 rpm. Finally, stop solution was added to the wells, and the OD value was read at 450 nm.

### Data collection

The basic clinical characteristics of sepsis were collected from electronic medical records, which included demographic characteristics, medical history, primary infection site, and biochemical index. The primary infection organism was also recorded after blood cultivation. Besides, the severity of sepsis was assessed within 24 after admission using acute physiology and chronic health evaluation II (APACHE II) score and sequential organ failure assessment (SOFA). All sepsis patients were followed up to 28 days or death, and 28-day mortality was recorded.

### Statistical analysis

SPSS 24.0 statistical software (IBM, Chicago, USA) was used for data analysis, and GraphPad Prism 7.02 (GraphPad Software Inc., San Diego, USA) was used for graph plotting. The normal distributed continuous variables were shown as mean with standard deviation (SD) (mean value ± SD), and skewed distributed or unknown distribution continuous variables were displayed as median with interquartile range (IQR) (median (1st quartile–3rd quartile)). Categorical variables were displayed as number with percentage (No. (%)). Comparison of variables between two groups was determined by the Wilcoxon rank sum test. Correlation between two continuous variables was analyzed by Spearman’s rank correlation test. Receiver operating characteristic (ROC) curve was plotted, and the area under curve (AUC) was used to assess the performance of variables in predicting 28-day mortality risk. Mortality risk factors were analyzed using forward stepwise multivariable logistic regression analysis. *P* value < 0.05 was considered as statistically significant.

## Results

### Basic clinical characteristics of sepsis patients

The total 125 sepsis patients had a mean age of 55.2 ± 12.6 years, among whom there were 45 (36.0%) females and 80 (64.0%) males (Table 1). The mean BMI was 23.4 ± 3.6 kg/m^2^. The numbers of patients who smoked and patients who drank were 43 (34.4%) and 51 (40.8%), respectively. As for controls, the mean values of age and BMI in controls were 53.9 ± 11.9 years and 22.7 ± 3.0 kg/m^2^, respectively. And the numbers of females and males in controls were 36 (36.0%) and 64 (64.0%), respectively. In addition, the numbers of controls who had smoking and controls with drinking history were 28 (28.0%), and 45 (45.0%), respectively. No difference was found between controls and sepsis patients regarding age (*P* = 0.426), gender (*P* = 1.000), BMI (*P* = 0.126), smoking status (*P* = 0.305), or drinking status (*P* = 0.527). Besides, the numbers of patients with primary infection site of abdominal infection, respiratory infection, skin and soft tissue infection, blood stream infection, central nervous system (CNS) infection, and other infections were 52 (41.6%), 27 (21.6%), 23 (18.4%), 13 (10.4%), 6 (4.8%), and 4 (3.2%), respectively. In addition, the primary organism was G− bacteria, G+ bacteria, anaerobes, fungus, and mycoplasmas in 66 (52.8%), 31 (24.8%), 17 (13.6%), 9 (7.2%), and 6 (4.8%) patients, respectively. The median values of APACHE II score and SOFA score were 12.0 (7.0–16.0) and 5.0 (4.0–8.0), respectively. Remaining information about the history of complications and laboratory indexes was displayed in Table 1.

**Table 1:** Characteristics of sepsis patients

Items	Controls (*N* = 100)	Sepsis patients (*N* = 125)	*P* value
Age (years), mean ± SD	53.9 ± 11.9	55.2 ± 12.6	0.426
Gender, No. (%)			1.000
Female	36 (36.0)	45 (36.0)	
Male	64 (64.0)	80 (64.0)	
BMI, (kg/m^2^), mean ± SD	22.7 ± 3.0	23.4 ± 3.6	0.126
Smoke, No. (%)	28 (28.0)	43 (34.4)	0.305
Drink, No. (%)	45 (45.0)	51 (40.8)	0.527
History of hypertension, No. (%)	-	45 (36.0)	-
History of hyperlipidemia, No. (%)	-	20 (16.0)	-
History of diabetes, No. (%)	-	16 (12.8)	-
History of CKD, No. (%)	-	13 (10.4)	-
History of CCVDs, No. (%)	-	24 (19.2)	-
Primary infection site, No. (%)			
Abdominal infection	-	52 (41.6)	-
Respiratory infection	-	27 (21.6)	-
Skin and soft tissue infection	-	23 (18.4)	-
Blood stream infection	-	13 (10.4)	-
CNS infection	-	6 (4.8)	-
Other infections	-	4 (3.2)	-
Primary organism, No. (%)			
G− bacteria	-	66 (52.8)	-
G+ bacteria	-	31 (24.8)	-
Anaerobes	-	17 (13.6)	-
Fungus	-	9 (7.2)	-
Mycoplasmas	-	6 (4.8)	-
Total culture negative	-	25 (20.0)	-
Scr (mg/dL), median (IQR)	0.8 (0.7–1.0)	1.4 (1.0–2.2)	< 0.001
Albumin (g/L), median (IQR)	42.8 (39.1–47.4)	27.0 (21.8–38.2)	< 0.001
WBC (*10^9^/L), median (IQR)	6.4 (5.4–7.5)	16.5 (11.8–26.9)	< 0.001
CRP (mg/L), median (IQR)	3.7 (2.5–6.4)	100.5 (51.6–131.4)	< 0.001
APACHE II score, median (IQR)	-	12.0 (7.0–16.0)	-
SOFA score, median (IQR)	-	5.0 (4.0–8.0)	-

*SD*, standard deviation; *BMI*, body mass index; *CKD*, chronic kidney disease; *CCVDs*, cardio-cerebrovascular diseases; *CNS*, central nervous system; *G−*, gram negative; *G+*, gram positive; *Scr*, serum creatinine; *IQR*, interquartile range; *WBC*, white blood cell; *CRP*, C-reactive protein; *APACHE II*, acute physiology and chronic health evaluation II; *SOFA*, sequential organ failure assessment

### JKAP level, Th1, and Th17 cell proportions in sepsis patients and controls

**Fig. 1:**
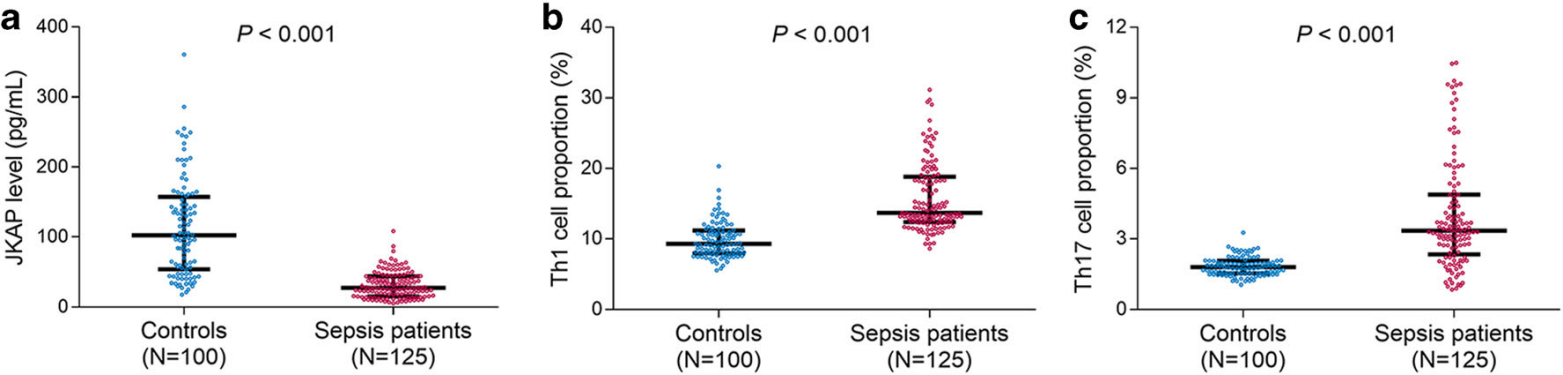
Comparisons of JKAP level, Th1, and Th17 cell proportions between sepsis patients and controls. The comparisons of JKAP level (**a**), Th1 cell proportion (**b**), and Th17 cell proportion (**c**) between sepsis patients and controls. The bars stood for 1st quartile, median, and 3rd quartile of JKAP level, Th1 cell proportion, or Th17 cell proportion, respectively. JKAP, Jun N-terminal kinase (JNK) pathway-associated phosphatase; Th1, T helper type 1; Th17, T helper type 17

The JKAP level (*P* < 0.001) (Fig. 1a) was decreased in sepsis patients than that in controls; however, Th1 cell proportion (*P* < 0.001) (Fig. 1b) and Th17 cell proportion (*P* < 0.001) (Fig. 1c) were increased in sepsis patients compared with controls.

### Correlation between JKAP level and Th1 or Th17 cell proportion

**Fig. 2:**
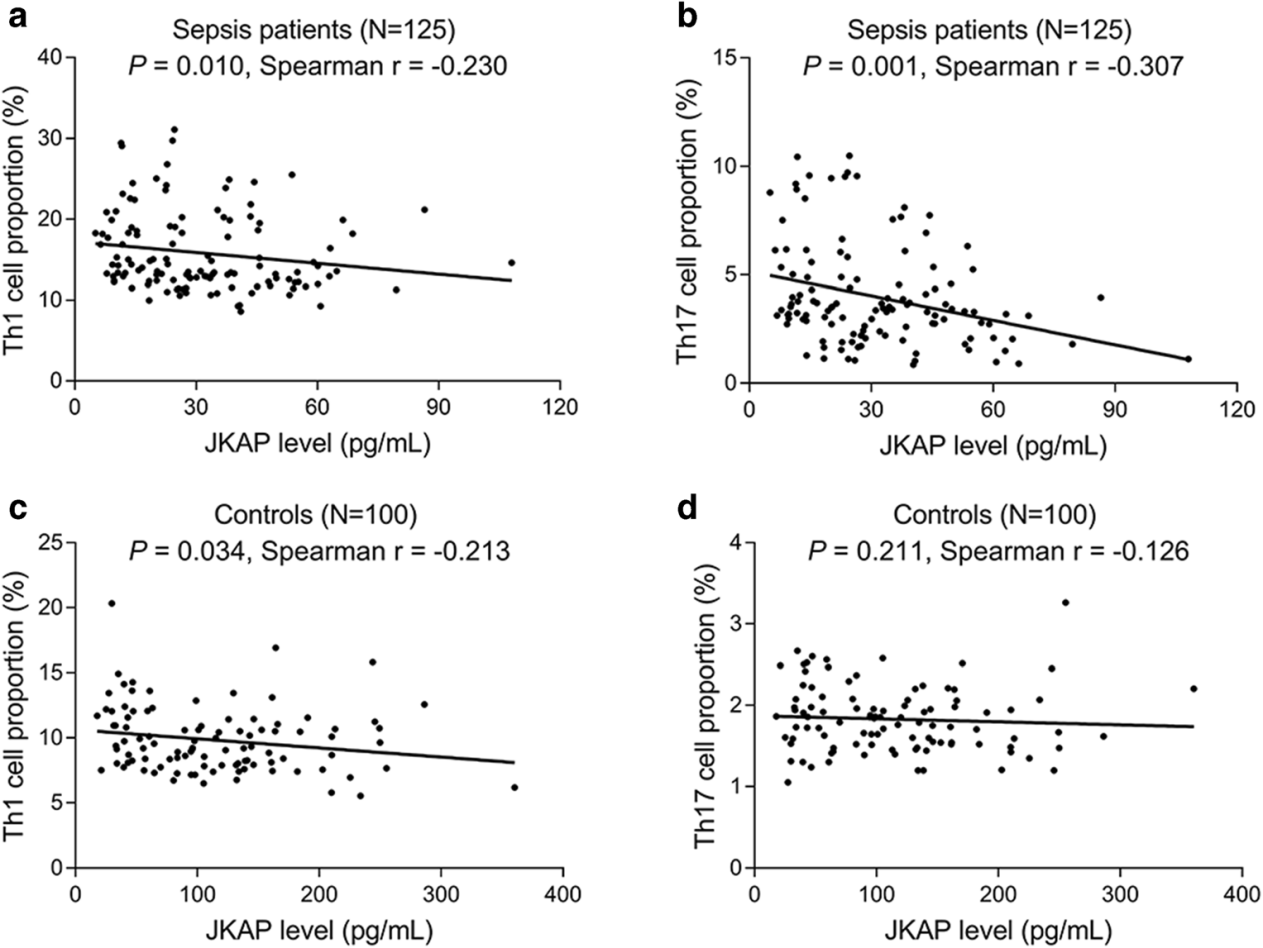
Analysis of association between JKAP level and Th1 or Th17 cell proportion. The association of JKAP level with Th1 cell proportion (**a**) or Th17 cell proportion (**b**) in sepsis patients, the association of JKAP level with Th1 cell proportion (**c**) or Th17 cell proportion (**d**) in controls. JKAP, Jun N-terminal kinase (JNK) pathway-associated phosphatase; Th1, T helper type 1; Th17, T helper type 17

In sepsis patients, JKAP level negatively associated with Th1 cell proportion (*P* = 0.010) (Fig. 2a) and Th17 cell proportion (*P* = 0.001) (Fig. 2b). In controls, JKAP level negatively associated with Th1 cell proportion (*P* = 0.034) (Fig. 2c); however, it did not associate with Th17 cell proportion (*P* = 0.211) (Fig. 2d).

### Correlation between JKAP level and inflammatory cytokines, APACHE II score, or SOFA score

**Fig. 3:**
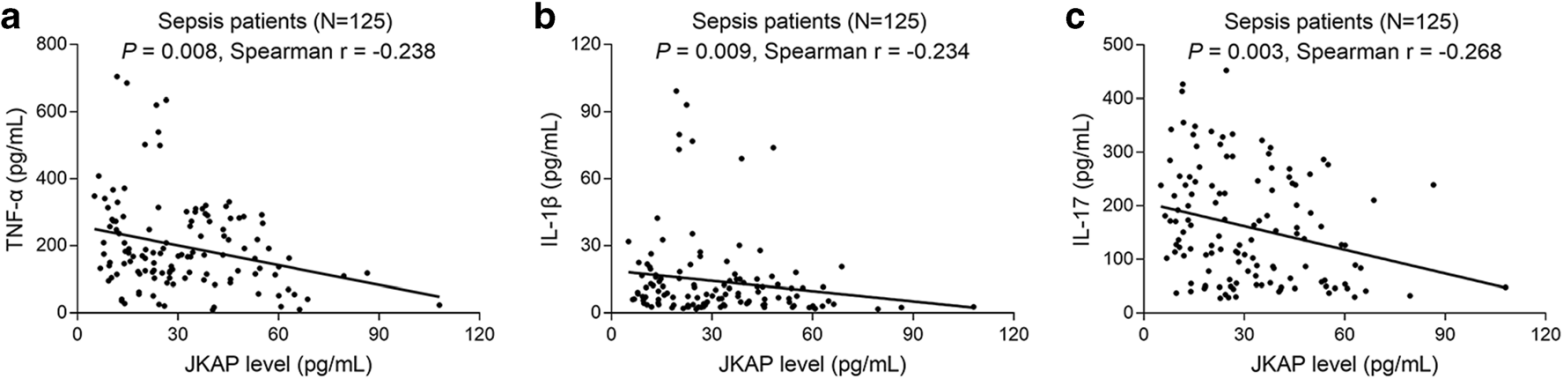
Analysis of association between JKAP level and inflammatory cytokines expressions in sepsis patients. The association of JKAP level with TNF-α (**a**), IL-1β (**b**), or IL-17 (**c**) expression in sepsis patients. JKAP, Jun N-terminal kinase (JNK) pathway-associated phosphatase; TNF-α, tumor necrosis factor-α; IL-1β, interleukin-1β; IL-17, interleukin-17

**Fig. 4:**
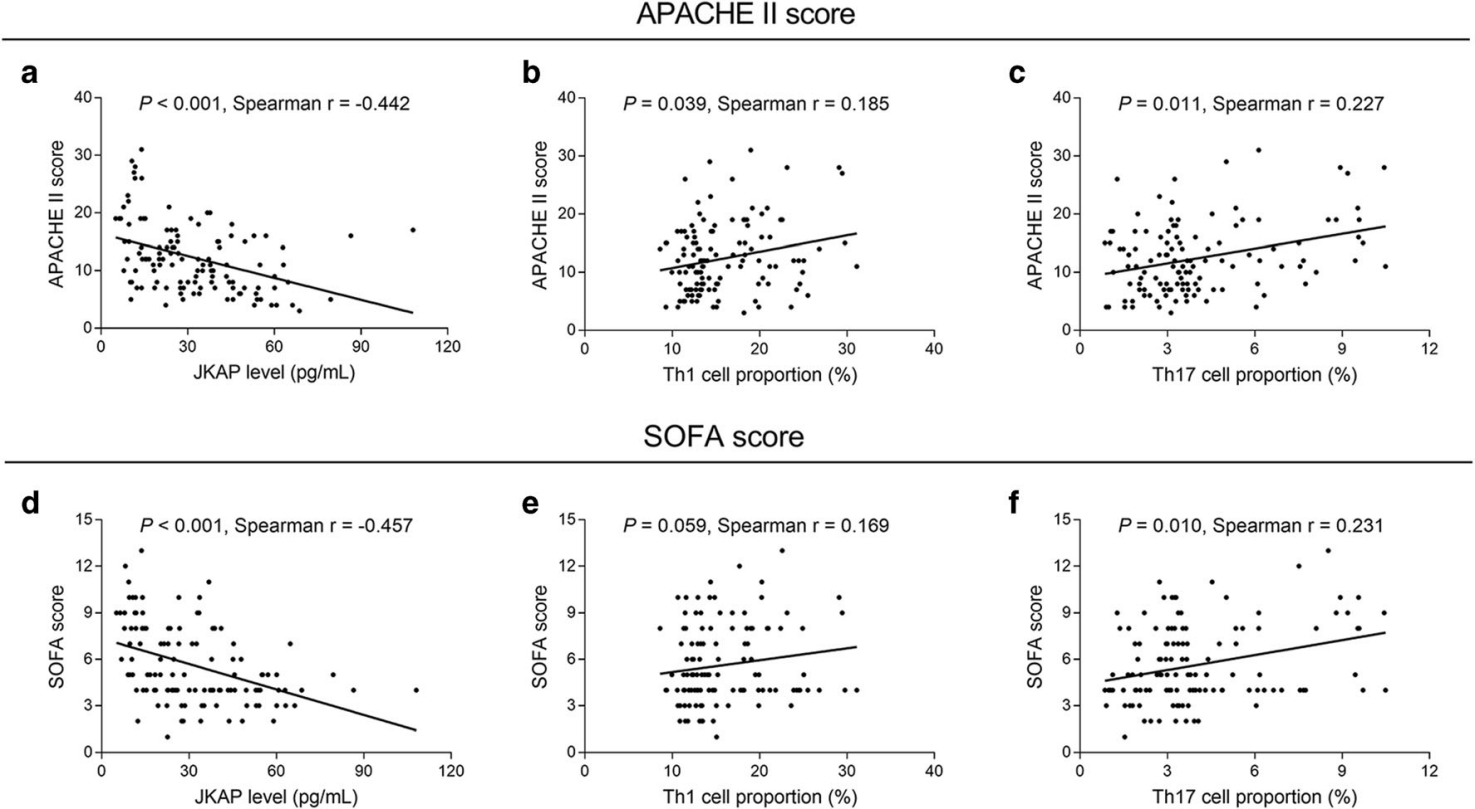
Analysis of association of JKAP level, Th1, or Th17 cell proportion with APACHE II or SOFA score in sepsis patients. Association between JKAP level (A), Th1 cell proportion (B), Th17 cell proportion (C) and APACHE II score, association between JKAP level (D), Th1 cell proportion (E), Th17 cell proportion (F) and SOFA score in sepsis patients. JKAP, Jun N-terminal kinase (JNK) pathway-associated phosphatase; Th1, T helper type 1; Th17, T helper type 17; APACHE II, acute physiology and chronic health evaluation II; SOFA, sequential organ failure assessment

In sepsis patients, regarding inflammatory cytokines, JKAP level negatively associated with TNF-α (*P* = 0.008) (Fig. 3a), IL-1β (*P* = 0.009) (Fig. 3b), and IL-17 (*P* = 0.003) (Fig. 3c) expressions. With regard to the indexes for assessing sepsis severity, JKAP level (*P* < 0.001) (Fig. 4A) negatively while Th1 cell proportion (*P* = 0.039) (Fig. 4B) and Th17 cell proportion (*P* = 0.011) (Fig. 4C) positively associated with APACHE II score. Besides, JKAP level (*P* < 0.001) (Fig. 4D) was negatively, but Th17 cell proportion (*P* = 0.010) (Fig. 4F) was positively associated with SOFA score; however, Th1 cell proportion (*P* = 0.059) (Fig. 4E) was not correlated with SOFA score.

### JKAP level in deaths and survivors

**Fig. 5:**
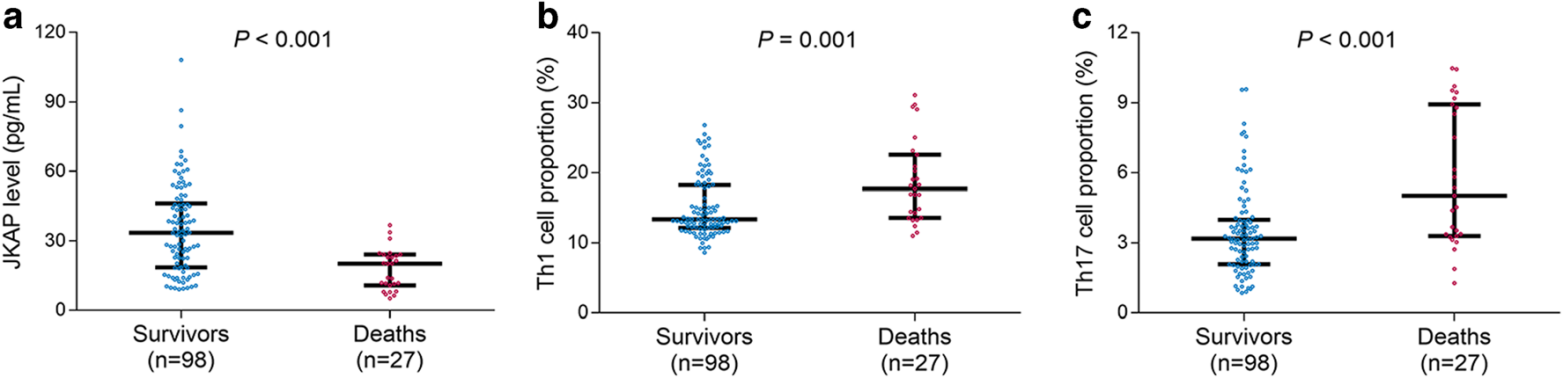
Comparison of JKAP level and Th1 or Th17 cell proportion in deaths and survivors. The comparison of JKAP level (**a**), Th1 cell proportion (**b**), or Th17 cell proportion (**c**) between deaths and survivors in sepsis patients. JKAP, Jun N-terminal kinase (JNK) pathway-associated phosphatase; Th1, T helper type 1; Th17, T helper type 17

**Fig. 6:**
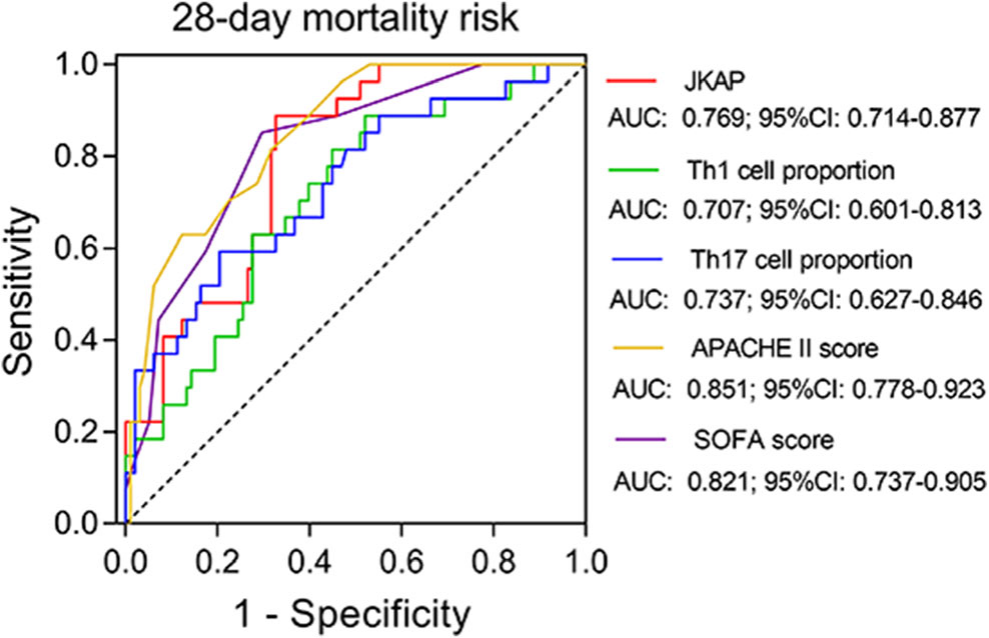
ROC analysis of JKAP level, Th1 or Th17 cell proportion, APACHE II score, and SOFA score for predicting 28-day mortality. ROC, receiver operating characteristic; JKAP, Jun N-terminal kinase (JNK) pathway-associated phosphatase; Th1, T helper type 1; Th17, T helper type 17; APACHE II, acute physiology and chronic health evaluation II; SOFA, sequential organ failure assessment; AUC, area under curve; 95% CI, 95% confidence interval

In sepsis patients, JKAP level (*P* < 0.001) (Fig. 5a) was downregulated in deaths compared with survivors, while Th1 cell proportion (*P* = 0.001) (Fig. 5b) and Th17 cell proportion (*P* < 0.001) (Fig. 5c) were upregulated in deaths than those in survivors. Then, the ROC curve showed that JKAP level presented with good value in differentiating deaths from survivors in sepsis patients; the AUC was 0.769 (95% CI 0.714–0.877) (Fig. 6). Besides, further ROC curve analyses revealed that Th17 cell proportion, APACHE II score, and SOFA score could also differentiated deaths from survivors with an AUC of 0.707 (95% CI 0.601–0.813), 0.737 (95% CI 0.627–0.846), 0.851 (95% CI 0.778–0.923), and 0.821 (95% CI 0.737–0.905), respectively.

### Independent predictive factors for mortality of sepsis patients

**Fig. 7:**
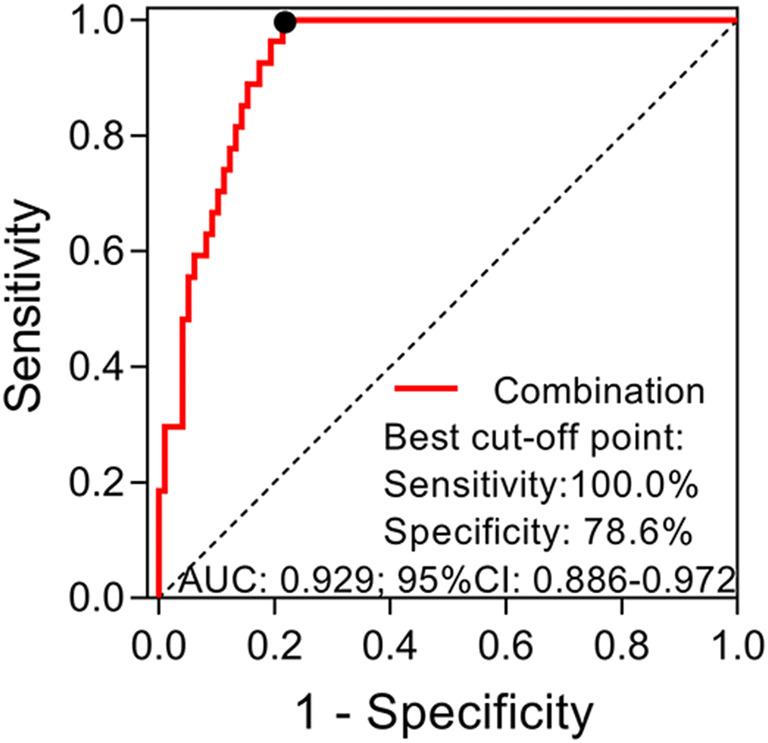
ROC analysis of the combination of independent predictive factors for 28-day mortality in sepsis patients. Dot in black stood for the best cut-off point. ROC, receiver operating characteristic; AUC, area under curve; 95% CI, 95% confidence interval

Forward stepwise multivariate logistic regression model revealed that JKAP level (*P* = 0.018, OR = 0.929) and Th17 cell proportion (*P* = 0.022, OR = 1.312) were independent factors for predicting 28-day mortality risk in sepsis patients (Table 2). In addition, age (*P* = 0.009, OR = 1.076) and APACHE II score (*P* = 0.002, OR = 1.203) were also independent 28-day mortality risk factors in sepsis patients. Afterward, further analysis disclosed that combination of the independent predictive factors for mortality, including JKAP level, Th17 cell proportion, age, and APACHE II score had great value in differentiating deaths from survivors with an AUC of 0.929 (95% CI 0.886–0.972) in sepsis patients (Fig. 7).

**Table 2:** Analysis of mortality risk factors

Items	Forward stepwise logistic regression model			
	*P* value	OR	95%CI	
			Lower	Higher
JKAP	0.018	0.929	0.874	0.988
Th17 cell proportion	0.022	1.312	1.040	1.655
Age	0.009	1.076	1.019	1.137
APACHE II score	0.002	1.203	1.068	1.356

*OR*, odds ratio; *CI*, confidence interval; *JKAP*, JNK pathway-associated phosphatase; *APACHE II*, acute physiology and chronic health evaluation II

## Discussion

Sepsis remains to be a major cause of health care burden around the world; besides its dreadful mortality, sepsis also leads to multiple clinical sequelae in those who survive, among which most of the sequelae are also fatal, such as the cardiovascular diseases [17–19]. This situation has promoted the investigation of underlying biological mechanisms of sepsis, which lastly aim at finding more targets for therapy. An imbalanced homeostasis is a critical feature of sepsis, which is resulted from a failure of the fight against infection in the immune system that finally causes perplexing interactions among host, infection, clinical presentations, clinical sequelae, and therapies [3]. Immune cell is a crucial component in the complex interactions inside sepsis patients; many types of T cells are involved in pathogenesis of sepsis [7]. In the present study, the JKAP level, Th1 cell proportion, Th17 cell proportion were detected, and their correlations with disease severity, inflammation level, and prognosis were assessed in sepsis patients. Our data showed that (i) JKAP was downregulated but Th1 and Th17 cell proportions were upregulated in sepsis patients compared with controls, and JKAP was negatively correlated with Th1 and Th17 cell proportions in sepsis patients; (ii) JKAP was negatively associated with pro-inflammatory cytokines and disease severity in sepsis patients; (iii) JKAP level and Th17 cell proportion were independent predictive factors for 28-day mortality in sepsis patients.

JKAP is essential in regulating the activities of JNK kinase, while recent studies have revealed other roles of JKAP, such as in sepsis-related biological processes, inflammation, and immunity. A previous study reports that in serum of patients with rheumatoid arthritis, the hypomethylated regions in DUSP22 (another name of JKAP) gene and CYP2E1 gene are correlated with more active and erosive disease condition [20]. In another study, serum JKAP expression is declined in Crohn’s disease (CD) patients compared with healthy individuals, and its overexpression associates with lower CD risk, activity, pro-inflammatory cytokines levels; more interestingly, serum JKAP overexpression is also an independent factor for predicting unfavorable response to TNF-α inhibitor in CD patients [7]. Similarly to the studies mentioned above, a study elucidates that JKAP in peripheral blood T cells is downregulated in SLE patients compared with healthy individuals, and decreased JAKP expression also correlates with higher disease activity index, anti-dsDNA antibody level, and worse clinical outcomes in SLE patients [15]. These studies indicate that JKAP could function as a biomarker in the management of several inflammation/immune-related diseases. As for sepsis, a former study reveals that a lower serum JKAP level is associated with reduced sepsis risk, more severe disease, elevated inflammation level, and an unfavorable survival profile [9]. These results are partially in line with ours. However, the previous study mainly aims at evaluating the role of JKAP in sepsis, while our study predominantly aimed at investigating the correlation between JKAP with Th1 cell or Th17 cell and their values in sepsis. In our study, we found that downregulated JKAP correlated with increased inflammation level, higher disease severity, and higher risk of 28-day mortality; these findings may be related to the role of JKAP in maintaining the normal immune response such as (1) repressing the CD4^+^ T cell activation and differentiation to Th1 cell as well as Th17 cell, which subsequently reduces inflammation [14]. (2) JKAP may also presents with protective effect in sepsis through diminishing the damage in organs, for instance, reducing the risk of developing nephritis as reported in a previous study conducted in SLE [15].

T cell governs the cell-mediated immunity in human body; when homeostasis is broken in internal environment, T cells may function abnormally and result in diseases. Th1 cell and Th17 cell are two relatively established cells that play critical roles in sepsis. For instance, a study illustrates that Th1, Th2, and Th17 type cytokines, IL-6, IL-8, and IL-10 are increased but IL-12 is decreased in patients with sepsis than healthy individuals; IL-8 overexpression correlates with renal and cardiac function damage, and IL-10 is downregulated in survivors than deaths [21]. Moreover, JKAP is involved in the regulation of Th cell functions. For example, in IBD, JKAP inhibits the activation of CD4^+^ T cells and represses the cell differentiation of Th1 and Th17 cells [14]. In addition, JKAP suppresses T cell receptor signaling and autoimmunity via inactivating Lck through dephosphorylating tyrosine-394 residue [22]. These studies reveal a negative modulatory role of JKAP for Th1 and Th17 cell functions. However, related studies are very limited; the correlation of JKAP with Th1 or Th17 cell proportion should be investigated in more studies. In this study, we found that Th1 and Th17 cell proportions were upregulated in sepsis patients compared with controls; meanwhile, JKAP was negatively associated with Th1 and Th17 cell proportions, and the latter ones were correlated with elevated disease severity in sepsis patients. Here are several possible explanations to these results: (1) JKAP is engaged in the negative regulation of Th1 and Th17 cell functions via coworking with other factors, for instance, interacting with the tyrosine-394 residue [14, 22]. This might result in the negative association of JKAP with Th1 and Th17 cell proportions in sepsis patients. However, the modulatory role of JKAP on Th1 and Th17 cell functions should be assessed by more animal or cell experiments. (2) As for the elevation of Th1 and Th17 proportions and their correlations with more severe disease in sepsis patients, it is reasonable because Th1 and Th17 cells play a role that promotes the disease progression in sepsis as reported by the previous studies [21, 23–25].

In addition, in the present study, JKAP level and Th17 cell proportion were found to be independent predictive factors for 28-day mortality, and combination of JKAP, Th17, age, and APACHE II score was of great value in predicting the risk of 28-day mortality in the present study. The predictive value of JKAP expression and Th17 cell proportion might be related to their functions that JKAP contributes to the conservation of homeostasis of immunity in sepsis, while Th17 functions as a promotor of sepsis progression [14, 24].

In the present study, several limitations existed. First, the sample size of 125 sepsis patients and 100 controls was relatively insufficient. We would like to enlarge the sample size; however, the sepsis patients were difficult to enroll due to lacking cases. Second, the sepsis patients were followed up to 28 days which was a relatively short follow-up period that should be prolonged in the future.

Collectively, blood JKAP correlates with decreased Th1 and Th17 cells, also associates with reduced inflammatory cytokines, disease severity, and favorable outcome in sepsis patients.
